# Depression among people living with type 2 diabetes in an urbanizing community of Nepal

**DOI:** 10.1371/journal.pone.0218119

**Published:** 2019-06-10

**Authors:** Avinash K. Sunny, Vijay K. Khanal, Ram B. Sah, Anup Ghimire

**Affiliations:** School of Public Health and Community Medicine, B.P Koirala Institute of Health Sciences, Dharan, Nepal; VU medisch centrum School of Medical Sciences, NETHERLANDS

## Abstract

**Background:**

Diabetes mellitus is a major public health problem which accounts for serious medical and economic consequences. Depression is an important associated condition that upsets the management and complications of diabetes. This study aimed to measure the prevalence of depression among people living with Type 2 Diabetes and to examine the factors linked with it.

**Methods:**

This community based, cross-sectional study was conducted among 278 people living with type 2 diabetes in Duhabi-Bhaluwa municipality, Nepal. A face to face interview was conducted using a pre-tested semi-structured questionnaire to gain information on socio-demographic characteristics and clinical profile of the participants. Depression was assessed using the Beck Depression Inventory (BDI-II) scale. Variables were categorical and were thus, compared with Pearson’s chi-square tests and binary logistic regression models.

**Results:**

The prevalence of depression in this study was 22.7%. Most people indicative of having depression were of older age, females, below secondary level education, with a smaller family size, with low family income, using insulin, without a family history of diabetes and/or having an additional illness. However, multivariate analysis showed that the only significant factors for depression were older age, education below secondary level, homemaker, smaller family size, using insulin and having an additional illness.

**Conclusion:**

The prevalence of depression in this study is consistent with that reported by other communities. Factors like older age, lower education level, being a homemaker, living in a small size family, insulin use and additional illness could increase the likelihood of developing Major Depressive disorder among people with type 2 diabetes, hence, psychosocial assessment is necessary along with diabetes management plan in a primary health care setting.

## Background

Diabetes mellitus is now rapidly rising as a worldwide epidemic. It has been projected to be the seventh most important cause of death worldwide by 2030 [1]. The diabetes population has rapidly rose from 108 million in 1980 to 422 million in 2014 globally and the predominance is seen mostly in low and middle-income countries [2]. In South-east Asia, it is estimated that 75 million people were living with diabetes in 2014 and this figure will rise to 123 million by 2035 [3]. In Nepal, there were 700,700 cases of diabetes in 2014. The estimated prevalence of diabetes for age group (20–79 years) was 4.6% with more than 14,778 deaths that year [3]. Studies conducted in Nepal have also found a growing diabetes prevalence of 25.9% in the elderly [4] and in a semi-urban population, the prevalence was 9.5% with a greater percentage in men (11.8%) than in women (7.9%) [5]. In Eastern Nepal, there were 6.3% of people living with diabetes [6].

Depression is another highly prevalent condition in the world. Globally, there are around 350 million people of all ages suffering from depression [7]. It is one of the major comorbid conditions associated with chronic diseases like diabetes [8,9]. The presence of depression is twice as more common in people with type 2 diabetes than in those without [10]. The role of psychopathology in patients with type 2 diabetes needs to be considered in order to achieve diabetes treatment goals for better health benefits [11].

Studies worldwide have pondered into the relationship between diabetes and depression. A systematic review which was conducted to examine the relationship of depression and type 2 diabetes reported that depression is associated with a 60% increase in the risk of developing type 2 diabetes [12]. In a systematic review to assess the prevalence of clinical depression in type 2 diabetes, it was found that the prevalence was significantly higher among patients with type 2 diabetes (17.6%) compared to those without diabetes (9.8%) [13]. A survey conducted in the US reported that 20% of type 2 diabetes population with depression were undiagnosed [14]. Similarly, a study among Greek adults reported of elevated depressive symptoms in 33.4% of the type 2 diabetes population [15]. There are similar reports in South East Asia as well. In Bangladesh, a population-based study indicated depressive symptoms among 29% of men and 30.5% of women who were newly diagnosed with type 2 diabetes [16]. In Pakistan, a study in rural area reported a depression prevalence of 14.7% in type 2 diabetic people [17]. In India, hospital studies have documented that the prevalence of depression among type 2 diabetic patients ranges from 8.5% to 32.5% with various scales [18]. Hospital-based studies of Nepal have reported a high prevalence of depression among type 2 diabetic patients of 40.3%, 44.1% and 54.1% [19–21].

In Nepal, poor mental health has been driven by many key risk factors such as poverty, conflict, displacement, gender discrimination, caste/ethnicity, unemployment, and (labor) migration [22]. Likewise in India, the risk factors associated with depression among people with type 2 diabetes are age, obesity, increased pill burden and diabetic complications [23]. Similarly, in Pakistan, the risk factors for depression among type 2 diabetes patients were living in a nuclear family, obesity, marital status, and the risk was high in people with the history of smoking, high blood pressure and gestational diabetes [24]. Poor glycemic control and insulin use are other risk factors found in studies in Bangladesh [16,25]. Thus, this suggests that there is a need for additional research to further correlate between these two comorbid conditions.

## Methods

### Aim, design, and setting

This cross-sectional study was done to assess the prevalence of depression among people living with type 2 Diabetes mellitus and to identify the factors associated with depression among them. This was a community-based study conducted in a semi-urban area of Duhabi-Bhaluwa, a municipality recently upgraded from a village development committee situated in the industrial corridor area of Sunsari district in eastern Nepal with a population of over 25,545 [26]. There is one health post (recently upgraded from the sub-health post) and few private clinics in the area. The study was conducted from September 2015 to August 2016.

### Study participants

This study included a purposive sample of people living with type 2 Diabetes mellitus. The inclusion criteria were: 1) males and females 20 years old and above; 2) self-report of being diagnosed with type 2 diabetes by a health care provider; and 3) willingness to participate in this study. The exclusion criteria were based on self-report of: 1) physical and or mental conditions that interfere with participation; 2) pregnancy and 3) psychiatric history or other mental illness.

### Sample size

A similar study conducted in Kathmandu, Nepal at tertiary care centers, reported the overall prevalence of depression among people living with type 2 diabetes mellitus is 40.3% [19]. So, using this prevalence with an allowable error of 15% at a confidence interval of 95%, the sample size was estimated to be 278 participants after adding a non-response rate of 10%.

### Instruments

Two sets of pretested semi-structured questionnaires were used for the study which are mentioned below.

### Socio-demographic and clinical questionnaire

A questionnaire was designed based on WHO NCD Steps Survey Nepal which was validated by letting our NCD experts read through the questionnaire and pretested among 30 respondents (10.8% of the estimated sample population), to gain information on

- Demographic factors such as age, gender, caste/ ethnicity, religion, marital status, type of family, family size, level of education, occupation and family income. Behavioral measurements such as personal history of smoking, alcohol use, and physical activity.
- Anthropometric measurement of height and weight.
- Medical history such as duration of illness, number of anti-diabetic medications, number of additional illness and family history of diabetes.

#### Beck Depression Inventory score questionnaire

The presence of depression was evaluated using the Beck Depression Inventory-Second Edition (BDI-II) [27]. The term “depression” in this study was referred to as the depressive symptoms identified in the BDI-II. The format of the BDI-II test is for the participants to answer one of the four phrases listed that best describes their state in the past two weeks including the day the questionnaire was interviewed. The most commonly used cut-off scores for BDI of ≥16 to indicate clinical depression was used in this study [28,29]. The BDI-II scale is validated for use in Nepal and it was translated to Nepali and back-translated into English for test-retest reliability [30]. The Cronbach’s Alpha based on standardized items when tested for all the 21 items on the BDI-II scale showed a good consistency of 0.76.

### The procedure of data collection

Door to door survey with the help of the health personnel such as local Female Community Health Volunteers, health assistant, and others from the local pharmacies, clinics and hospitals was done for people living with type 2 diabetes mellitus. They were identified from their medical prescription record and their medical history. The procedure and purpose of the study were explained and participants were recruited based on inclusion and exclusion criteria. Those willing to participate were either interviewed in their respective homes or nearby health facility at their time of convenience. An informed consent was read to the participants and once signed, a face to face interview was conducted using the pretested semi-structured questionnaires required for the study. If the person was not present at the time of the interview, a second visit was made to the same household. If despite this, the participants were not present, another household was taken to complete the estimated sample size. Participants were recruited until the sample size was met.

### Data management and statistical analysis

The collected data was entered into Microsoft Excel and coded with alphanumerical codes which were finally converted into Statistical Package for Social Sciences (SPSS) for statistical analysis. Continuous variables were categorized using their median value. For categorizing family size, we used the median of the total members in the family of the respondents. Bivariate analysis was carried out by applying Chi-square (χ2) tests to compare the factors associated with depression scores ≥ 16 on BDI-II at 95% Confidence Interval (CI) where p was considered to be significant at <0.05. The variables that were significant at p < 0.2 from the bivariate analysis were considered for multivariate analysis using multiple logistic regression where backward LR method was specified in order to identify the factors associated with depression among people living with type 2 diabetes mellitus.

### Ethical consideration

The study was approved by the Institutional Review Committee of B P Koirala Institute of Health Sciences (Reference number: 344/071/072-IRC). Written informed consent was obtained from the participants before data collection and all information were kept confidential.

## Results

A total of 278 participants were recruited in the study. There were no non-response and 10 households with type 2 diabetes were not present at the time of interview. They were revisited and everyone responded on their second visit. The study population was aged between 32 and 82 years (mean ± SD = 54.3 ± 11.2) with more male participants (67.6%). Most of the participants (70.5%) in this study belonged to joint family and the median family size in this study was 7 members (IQR = 5–9) ranging from 1 to 18 members. The prevalence of depression in this study was found to be 22.7%. Table 1 shows the bivariate analysis of the factors associated with depression in this study. Variables such as age, gender, marital status, educational status, work status, ethnicity, family income, family size, number of OHA (Oral hypoglycemic agents), family history of diabetes, insulin use and additional illness were found to be significantly associated with depression in the bivariate model.

**Table 1:** Bivariate analysis of the factors associated with depression (n = 278).

Variable	Total	Depression (BDI≥16)	No Depression (BDI<16)	p-value
Age				
20–40	33(11.9%)	2(3.2%)	31(14.4%)	0.019
40–60	170(61.2%)	38(60.3%)	132(61.4%)	
≥60	75(27.0%)	23(36.5%)	52(24.2%)	
Gender				
Male	188(67.6%)	36(57.1%)	152(70.7%)	0.043
Female	90(32.4%)	27(42.9%)	63(29.3%)	
Religion				
Hindu	232(83.5%)	50(79.4%)	182(84.7%)	0.321
Others	46(16.5%)	13(20.6%)	33(15.3%)	
Marital status				
Married	255(91.7%)	54(85.7%)	201(93.5%)	0.049
Others	23(8.3%)	9(14.3%)	14(6.5%)	
Ethnicity				
Dalit	28(10.1%)	3(4.7%)	25(11.6%)	0.157
Janjati	32(11.5%)	9(14.3%)	23(10.7%)	
Madhesi	155(55.8%)	38(60.3%)	117(54.4%)	
Muslim	39(14.0%)	11(17.5%)	28(13.0%)	
Brahmin/Chhetri	24(8.6%)	2(3.2%)	22(10.2%)	
Education				
Below Secondary	188(67.6%)	58(92.1%)	130(60.5%)	0.001
Secondary and above	80(32.4%)	5(7.9%)	85(39.5%)	
Work status				
Employed	167(60.0%)	27(42.9%)	140(65.1%)	0.004
Homemaker	68(24.5%)	24(38.1%)	44(20.5%)	
Retired	43(15.5%)	12(19.0%)	31(14.4%)	
Family type				
Nuclear	82(29.5%)	17(27.0%)	65(30.2%)	0.619
Joint	196(70.5%)	46(73.0%)	150(69.8%)	
Family size				
≤7	164(59.0%)	44(69.8%)	120(55.8%)	0.047
>7	114(41.0%)	19(30.2%)	95(44.2%)	
Family income (NPR monthly)				
≤30000	141(50.7%)	39(61.9%)	102(47.4%)	0.043
>30000	137(49.3%)	24(38.1%)	113(52.6%)	
Smoking				
Yes	39(14.0%)	10(15.9%)	29(13.5%)	0.632
No	239(86.0%)	53(84.1%)	186(86.5%)	
Alcohol use				
Yes	66(23.7%)	12(19.0%)	54(25.1%)	0.319
No	112(76.3%)	51(81.0%)	161(74.9%)	
BMI				
<25	139(50.0%)	31(49.2%)	108(50.2%)	0.886
≥25	139(50.0%)	32(50.8%)	107(49.8%)	
Duration of illness				
≤5 years	133(47.8%)	26(41.3%)	107(49.8%)	0.235
>5 years	145(52.2%)	37(58.7%)	108(50.2%)	
Number of OHA				
None	49(17.6%)	6(9.5%)	43(20.0%)	0.119
One	127(45.7%)	34(54.0%)	93(43.3%)	
Two or more	102(36.7%)	23(36.5%)	79(36.7%)	
Insulin use				
Yes	23(8.3%)	14(22.2%)	9(4.2%)	0.001
No	255(91.7%)	49(77.8%)	206(95.8%)	
Family history of Diabetes				
Yes	101(36.3%)	14(22.2%)	87(40.5%)	0.008
No	177(63.7%)	49(77.8%)	128(59.5%)	
Number of additional illness				
None	136(48.9%)	20(31.7%)	116(54.0%)	0.007
One	119(42.8%)	35(55.6%)	84(39.1%)	
Two or more	23(8.3%)	8(12.7%)	15(7.0%)	

Table 2 shows the multivariable model where the variables significantly associated were age, work status, education level, family size, insulin use and additional illness. From the odds ratio evaluation, the odds of having depression among those in the age group of 60 years and above was 7.55 times more likely compared to those less than 40 years of age (p = 0.027). The probability of depression among homemakers was 6.15 times more likely compared to those who were employed (p = 0.024). The probability of depression among those with below secondary level education was 8.07 times more than those with education level above secondary (p<0.001). Similarly, the probability of depression among those living in a smaller family size with ≤7 members was 3.21 times more than those living in a family with 7 members (p = 0.002). The probability of depression was 9.11 times higher among who used insulin (p<0.001). When compared to those with no additional illness, the odds of having depression was 8.22 times higher with two or more additional illness (p = 0.002) whereas the odds of having depression was 2.42 times higher with one additional illness (p = 0.023).

**Table 2:** Multivariate analysis of the factors associated with depression (n = 278).

Variables	β coefficient	p-value	Odds Ratio with 95% C. I.
Age			
<40		0.086	Ref
40–60	1.568	0.061	4.79 (0.92–24.76)
≥60	2.021	0.027	7.55 (1.25–45.34)
Gender			
Male			Ref
Female	-1.488	0.058	0.22 (0.04–1.05)
Work status			
Employed		0.062	Ref
Homemaker	1.817	0.024	6.15 (1.27–29.74)
Retired	-.256	0.646	0.77 (0.26–2.30)
Education			
Above secondary			Ref
Below secondary	2.089	<0.001	8.07 (2.82–23.07)
Family size			
>7			Ref
≤7	1.169	0.002	3.21 (1.54–6.69)
Insulin Use			
No			Ref
Yes	2.210	<0.001	9.11 (2.98–27.81)
Number of Additional illness			
None		0.005	Ref
One	0.885	0.023	2.42 (1.12–5.20)
Two or more	2.107	0.002	8.22 (2.16–31.24)
Constant	-6.018	0.000	0.002

## Discussion

### Prevalence of depression

Among 278 participants included in the study, the prevalence of clinically defined depression (BDI ≥ 16) with the BDI-II score was found to be 22.7%. Similar prevalence of depression in people with type 2 diabetes was detailed in other published studies. A meta-analysis carried out in the United States showed that the prevalence was significantly higher in clinical (32%) than in community (20%) samples [10]. The rate is also similar to one noticed in the United States, where the prevalence of depression among individuals with type 2 diabetes was 24% [31]. A community study from Bangladesh showed a depression prevalence of 27.9% with diabetes [16]. Likewise, a study done in Jordan reported that the prevalence rate of undiagnosed depression among type 2 diabetic people was 20.1% [32]. The similar prevalence rate of depression of 24% was reported in a diabetic population study from South Australia [33]. The similarity in these rates could be possibly due to population samples surveyed in those studies. However, a large recent (2016) population based study on depression among type 2 diabetic citizens of United states estimated an overall prevalence of 10.6% which is quite lower than the estimated prevalence in this study [34]. This may be due to improvement in recognition and treatment plan for people with diabetes in recent years. The prevalence rate in this study is lower than that observed in other studies in Nepal of about 40.3%, 54.1% and 44% [19–21]. This could be because these studies were carried out in tertiary care settings of Kathmandu, the capital city of Nepal where the prevalence is expected to be high because of more sick people reporting to hospitals from all over the country. The difference in findings could also be due to the different scales of BDI and their cut off used. In this study, the cut off for depression with BDI-II ≥ 16 was used to indicate clinical depression while BDI-Ia ≥ 20 and BDI-II ≥ 14 were used in other similar studies in Nepal [19,20]. The prevalence in this study is also slightly higher compared to the depression prevalence (15%) estimated using BDI-Ia ≥ 20 in a hospital study of hypertensive patients in Nepal [35].

### Factors associated with depression among people with type 2 diabetes mellitus

Age has been significantly linked to depression in the general population of Nepal [22,36]. Higher chances of depression with increasing age group of the diabetic population suggests that the prevalence of depression in this study increased with increasing age. Various studies have found the association of age with depression among people living with diabetes with older age having more chances of depression [20,25,37]. This could be probably because an older adult may feel a loss of control over their lives due to limited financial resources, feelings of hopelessness and isolation that often provoke thoughts of suicide and emotions like sadness, anxiety, loneliness and low self-esteem, which in turn lead to social withdrawal and apathy. However, a hospital study from Kathmandu, Nepal did not find any association between age and depression among people living with diabetes [19]. This can be explained by better financial status and social involvement of the study population.

Education level was another significantly associated factor with depression in this study. Many other studies have reported the similar association of lower educational status with depression in diabetes population [20,29,32]. This association with lower educational status among people with type 2 diabetes can be linked to their lesser understanding of the disease severity, drug compliance and dietary regulation which makes them vulnerable to complications. Similarly, work status was significantly associated with depression with the probability of depression among homemaker being the most. This was expected since not having work is in itself a depressing factor and also since there is a financial burden imposed by the disease. Lack of employment was also reported in a similar study conducted in Palestine [29] and a homemaker, as a predictor of depression, has been reported by many studies [20,38].

Family size was a significant predictor of depression with the probability among those living in a smaller family size of ≤7 members being higher. This could be since many members in a family brings a feeling of support socially and economically which is required to cope up with the healthcare cost of living with diabetes. However, this finding was contrasting with another similar study conducted in Kathmandu, Nepal which is an urban area where few people are found living in a family [19]. Another significant association in this study was seen with insulin use which showed higher chances of depression (OR = 9.11) compared to those not using insulin. Despite the small number of people (23/278) using insulin in this study, it was highly significant. This could indicate insulin use being taken as a burden to their daily activities and is consistent with many studies [19,20,32]. A large-scale study of adults in the US also reported a higher rate of depression in people using insulin [31]. Insulin use is concluded as a depressing factor in many studies since it is perceived to be taken for greater disease severity [10,13,39,40].

The chances of depression were lower among people who reported of having a family history of diabetes when compared to those who were unaware or didn’t have a family history. A similar study found that lack of family history was associated with depression since it can help to reduce the stress and anxiety related to the disease and regularize the experience [19]. A study of African American adults found that people with a family history of diabetes were more aware of the related risk factors and engaged themselves in healthy behaviors compared to those who were unknown of the family history [41]. The likelihood of depression increased with the number of additional illness among people with diabetes in this study. This finding is consistent with the study done in Palestine which showed that depression was twice as likely among people with type 2 diabetes with two or more additional illness [29]. Similarly, studies conducted in Pakistan and Bangladesh also reported that additional illness to diabetes increases the risk of depression [25,37]. A meta-analysis also concluded that depression was five times more likely when people with diabetes were having an additional illness [42]. Having additional illness is seen to be an obvious burden to the existing illness.

### Limitations of the study

There are few limitations in this study. Firstly, the participant’s responses may be less reliable as it could have been a socially acceptable response. Another limitation is that this study could not assess the blood glucose level as a predictor to establish a relationship with depression. Several studies have found HbA1C as a glycemic control measure to be associated with depression. Finally, this study would underestimate the total prevalence of overall depression since people with a prior history of clinically diagnosed depression and those currently under anti-depressant medication were not included in the study.

## Conclusion

The prevalence of depression in this study is similar to other reported studies. Only significant factors for depression were older age, homemaker, education below the secondary level, smaller family size (≤7), insulin use and additional illness.

### Recommendations

Further studies with longitudinal design in the community settings are needed to highlight the stressors associated with the disease. There should be an approach to psychoeducate patients, their family members and health care workers to increase alertness on the risk of depression in people with type 2 diabetes. Psychosocial assessment in the diabetes management plan is recommended in a primary health care setting as well.
